# Acceptance and Perception of Artificial Intelligence Usability in Eye Care (APPRAISE) for Ophthalmologists: A Multinational Perspective

**DOI:** 10.3389/fmed.2022.875242

**Published:** 2022-10-13

**Authors:** Dinesh V. Gunasekeran, Feihui Zheng, Gilbert Y. S. Lim, Crystal C. Y. Chong, Shihao Zhang, Wei Yan Ng, Stuart Keel, Yifan Xiang, Ki Ho Park, Sang Jun Park, Aman Chandra, Lihteh Wu, J. Peter Campbel, Aaron Y. Lee, Pearse A. Keane, Alastair Denniston, Dennis S. C. Lam, Adrian T. Fung, Paul R. V. Chan, SriniVas R. Sadda, Anat Loewenstein, Andrzej Grzybowski, Kenneth C. S. Fong, Wei-chi Wu, Lucas M. Bachmann, Xiulan Zhang, Jason C. Yam, Carol Y. Cheung, Pear Pongsachareonnont, Paisan Ruamviboonsuk, Rajiv Raman, Taiji Sakamoto, Ranya Habash, Michael Girard, Dan Milea, Marcus Ang, Gavin S. W. Tan, Leopold Schmetterer, Ching-Yu Cheng, Ecosse Lamoureux, Haotian Lin, Peter van Wijngaarden, Tien Y. Wong, Daniel S. W. Ting

**Affiliations:** ^1^Singapore Eye Research Institute (SERI), Singapore National Eye Center (SNEC), Singapore, Singapore; ^2^School of Medicine, National University of Singapore (NUS), Singapore, Singapore; ^3^Duke-NUS Medical School, Singapore, Singapore; ^4^Department of Ophthalmology, University of Melbourne, Melbourne, VIC, Australia; ^5^State Key Laboratory of Ophthalmology, Zhongshan Ophthalmic Center (ZOC), Sun Yat-sen University, Guangzhou, China; ^6^Department of Ophthalmology, Seoul National University College of Medicine, Seoul, South Korea; ^7^Department of Ophthalmology, Seoul National University Bundang Hospital, Seongnam-si, South Korea; ^8^Department of Ophthalmology, Southend University Hospital, Southend-on-Sea, United Kingdom; ^9^Asociados de Macula, Vitreo y Retina de Costa Rica, San José, Costa Rica; ^10^Casey Eye Institute, Oregon Health and Science, Portland, OR, United States; ^11^Department of Ophthalmology, University of Washington, Seattle, WA, United States; ^12^Moorfields Eye Hospital, London, United Kingdom; ^13^Department of Ophthalmology, University Hospitals Birmingham NHS Foundation Trust, Birmingham, United Kingdom; ^14^Institute of Ophthalmology, University College London (UCL), London, United Kingdom; ^15^International Eye Research Institute of the Chinese University of Hong Kong (Shenzhen), Shenzhen, China; ^16^C-MER International Eye Research Center of the Chinese University of Hong Kong (Shenzhen), Shenzhen, China; ^17^Specialty of Clinical Ophthalmology and Eye Health, Faculty of Medicine and Health, Westmead Clinical School, The University of Sydney, Sydney, NSW, Australia; ^18^Department of Ophthalmology, Faculty of Medicine, Health and Human Sciences, Macquarie University Hospital, Sydney, NSW, Australia; ^19^Department of Ophthalmology, University of Illinois College of Medicine, Chicago, IL, United States; ^20^Department of Ophthalmology, Doheny Eye Institute, Los Angeles, CA, United States; ^21^Department of Ophthalmology, Tel Aviv Medical Center, Tel Aviv, Israel; ^22^Department of Ophthalmology, University of Warmia and Mazury, Olsztyn, Poland; ^23^Institute for Research in Ophthalmology, Ponzan, Poland; ^24^OasisEye Specialists, Kuala Lumpur, Malaysia; ^25^Department of Ophthalmology, Chang Gung Memorial Hospital, Taoyuan, Taiwan; ^26^Oculocare Medical AG, Zurich, Switzerland; ^27^Department of Ophthalmology and Visual Sciences, The Chinese University of Hong Kong (CUHK), Hong Kong, Hong Kong SAR, China; ^28^Vitreoretinal Research Unit, Department of Ophthalmology, Chulalongkorn University and King Chulalongkorn Memorial Hospital, Bangkok, Thailand; ^29^Department of Ophthalmology, College of Medicine, Rangsit University, Rajavithi Hospital, Bangkok, Thailand; ^30^Vitreo-Retinal Department, Sankara Nethralaya, Chennai, India; ^31^Department of Ophthalmology, Kagoshima University, Kagoshima, Japan; ^32^Bascom Palmar Eye Institute, Miami, FL, United States; ^33^Copenhagen University Hospital, Copenhagen, Denmark; ^34^Tsinghua Medicine, Tsinghua University, Beijing, China

**Keywords:** ophthalmology, artificial intelligence (AI), regulation, implementation, translation

## Abstract

**Background:**

Many artificial intelligence (AI) studies have focused on development of AI models, novel techniques, and reporting guidelines. However, little is understood about clinicians' perspectives of AI applications in medical fields including ophthalmology, particularly in light of recent regulatory guidelines. The aim for this study was to evaluate the perspectives of ophthalmologists regarding AI in 4 major eye conditions: diabetic retinopathy (DR), glaucoma, age-related macular degeneration (AMD) and cataract.

**Methods:**

This was a multi-national survey of ophthalmologists between March 1st, 2020 to February 29th, 2021 disseminated *via* the major global ophthalmology societies. The survey was designed based on microsystem, mesosystem and macrosystem questions, and the software as a medical device (SaMD) regulatory framework chaired by the Food and Drug Administration (FDA). Factors associated with AI adoption for ophthalmology analyzed with multivariable logistic regression random forest machine learning.

**Results:**

One thousand one hundred seventy-six ophthalmologists from 70 countries participated with a response rate ranging from 78.8 to 85.8% per question. Ophthalmologists were more willing to use AI as clinical assistive tools (88.1%, *n* = 890/1,010) especially those with over 20 years' experience (OR 3.70, 95% CI: 1.10–12.5, *p* = 0.035), as compared to clinical decision support tools (78.8%, *n* = 796/1,010) or diagnostic tools (64.5%, *n* = 651). A majority of Ophthalmologists felt that AI is most relevant to DR (78.2%), followed by glaucoma (70.7%), AMD (66.8%), and cataract (51.4%) detection. Many participants were confident their roles will not be replaced (68.2%, *n* = 632/927), and felt COVID-19 catalyzed willingness to adopt AI (80.9%, *n* = 750/927). Common barriers to implementation include medical liability from errors (72.5%, *n* = 672/927) whereas enablers include improving access (94.5%, *n* = 876/927). Machine learning modeling predicted acceptance from participant demographics with moderate to high accuracy, and area under the receiver operating curves of 0.63–0.83.

**Conclusion:**

Ophthalmologists are receptive to adopting AI as assistive tools for DR, glaucoma, and AMD. Furthermore, ML is a useful method that can be applied to evaluate predictive factors on clinical qualitative questionnaires. This study outlines actionable insights for future research and facilitation interventions to drive adoption and operationalization of AI tools for Ophthalmology.

## Introduction

Aging populations are fueling an exponential growth in the demand for eye care and insufficient capacity of eye care services in many health systems (1–3). This has created mounting pressure to develop solutions that optimize existing resources, facilitate the triage of patients, and expand the surge capacity of health systems (4, 5). These constraints were heightened by clinical service disruptions during the coronavirus disease 2019 (COVID-19) outbreak, ranging from operational reorganization for pandemic responses as well as a mounting backlog of postponed elective services (5, 6). In response, the medical community has identified artificial intelligence (AI) as a potential solution to mitigate these pressures. A mature implementable AI digital solution could provide scalable automation, alleviate resource bottlenecks and expedite treatment process. This is particularly relevant for Ophthalmology, where extensive use of digital sensors and image-acquisition technologies provide a strong foundation for AI deployment (7).

Currently, AI for automated classification in ophthalmic imaging has been validated with clinically acceptable performance and evaluated in many studies (8–15), including clinical trials (7, 16), health economic analyses (17), reporting standards such as CONSORT-AI, SPIRIT-AI, and STARD-AI (18–22), AI ethics, trust, reproducibility, and explainability (23, 24). However, expert consensus for the acceptable forms of clinical AI applications have not been established. In recent clinical AI implementation studies, a range of barriers were reported to hinder successful clinical translation, for example lack of trust amongst stakeholders, organizational lack of capacity, and system limitations in necessary supporting infrastructure (25).

Earlier studies have surveyed general perceptions of AI among different users (e.g., medical students, radiologists) (26–28), although sample sizes were relatively small and limited to specific society or geographical location. Furthermore, none have evaluated the entire healthcare ecosystem from the microsystem (e.g., individual practitioners) (29, 30), to mesosystem (e.g., specific organizations) and macrosystem (e.g., system-level policies and population screening services) (31). These are crucial steps to determine practical requirements for effective clinical implementation at each level of the health system, and to inform initiatives to facilitate sustained adoption (32). The objective of this study is to evaluate the acceptance and perception of AI applications among ophthalmologists for the leading causes of preventable blindness including diabetic retinopathy (DR), glaucoma, age-related macular degeneration (AMD), and/or cataract, using the United States Food and Drug Administration (US FDA) software as medical device (SaMD) guideline as a reference framework.

## Methods

This was an expert survey investigation of eye care practitioners regarding their perspectives for clinical artificial intelligence (AI) solutions in Ophthalmology. Responses from ophthalmologists to this anonymous web-based electronic survey are investigated in partnership with professional associations through convenient selection to reflect the spectrum of geographical regions and subspecialties across the Ophthalmology medical field. The temporal proximity of the study period (1 March 2020–1 March 2021) to the COVID-19 outbreak (declared a pandemic by the World Health Organization on 11 March 2020) also enabled collection of data regarding its impact on provider perspectives of AI applications. This research adhered to the tenets of the declaration of Helsinki, and Singhealth Institutional Review Board (IRB) approval was obtained with waiver of the need for informed consent (CIRB Ref 2020/2219).

### Survey Development

The study survey was iteratively refined through literature review to develop semi-structured dichotomous and Likert questions (Appendix 1). This was followed by a pilot exercise with 6 clinical and academic Ophthalmology experts in Singapore, China, and Australia who have extensive experience in the conduct of AI-related research and recently published an AI-related peer-reviewed manuscript. Based on the results of the pilot exercise, the survey was finalized with optional responses programmed for individual qualitative questions. This was to avoid forced responses in the event a question was irrelevant for a given participants' practice setting [e.g., for Supplementary Tables 1A–C, regions with a lack of trained allied primary eye care services (PECS) or primary care provider (PCP) with eye care services]. Research was conducted remotely during the COVID-19 pandemic. It was hosted on an online survey platform (SurveyMonkey, San Mateo, USA) and designed to assess ophthalmologists' perspectives regarding their own organizations willingness to adopt AI as well as their own professional acceptance of various clinical AI applications for eye care.

First, professional acceptance of various clinical AI applications for eye care services was evaluated based on the regulatory guidance outlined in the SAMD document prepared by the International Medical Device Regulators Forum (IMDRF) working group chaired by the US FDA (33). A risk-based approach is applied accordingly, with ophthalmologists responding about their acceptance of AI applications in a matrix questionnaire based on the intended user, clinical context, and significance of the information provided to the healthcare decision based on the SaMD framework.

Intended users included ophthalmologists, primary eye care providers (PECPs, such as optometrists and opticians) and primary care providers (PCPs) with eye care services. Clinical contexts evaluated include the detection of common eye diseases DR, glaucoma, AMD and/or cataract.

Significance of information provided to the healthcare decision were classified based on the SaMD framework for intended uses to inform clinical management, drive clinical management, or diagnose eye diseases, as assistive tool, clinical decision support (CDS) tool or diagnostic tool, respectively (33). Applications of AI as assistive tools to inform clinical management include highlighting areas of interest in ophthalmic images for the practitioners' consideration to arrive at a diagnosis and treatment plan. Applications of AI as CDS tools to drive clinical management include providing possible provisional diagnoses based on areas of interest in ophthalmic images for the practitioners' consideration to develop a treatment plan. Applications of AI as diagnostic tools include providing a clinical diagnosis including stage of disease based on ophthalmic images, with or without management recommendations.

Next, Ophthalmologists' views on factors contributing to AI acceptance were evaluated considering all levels of the healthcare ecosystem from the microsystem to the macrosystem. First, the factors contributing to technology acceptance at the level of the healthcare microsystem including professional acceptance of clinical AI applications, acceptable level of error, perceived impact on professional roles, and potential barriers/enablers for adoption were explored (31). Second, factors contributing to technology acceptance at the level of the healthcare mesosystem were explored, including perceived willingness to adopt AI for clinical services within the organizations they practice in, anticipated organizational impact of clinical AI adoption, and likelihood of organizational facilitation of its adoption. At the mesosystem level, participants were also asked about the perceived willingness of their organizations to adopt AI for screening or diagnosis of the four major contributors to avoidable blindness, namely, diabetic retinopathy (DR), glaucoma, age-related macular degeneration (AMD) or cataract (34). Furthermore, participants were asked to report any anticipated organizational impact of the adoption of clinical AI for eye care services. Third, participants were asked to report their perspectives on the potential value of AI at the level of the macrosystem for eye care services. Finally, given the proximity of survey dissemination with the onset of the coronavirus disease 2019 (COVID-19) pandemic, participants were also surveyed about their perceptions regarding its impact acting at the level of the healthcare macrosystem, on future meso- and micro-system priorities for adoption.

### Survey Dissemination

The web-based survey was disseminated through snow-ball sampling of professional Ophthalmology associations. Collaborating associations were selected to represent participants from a breadth of clinical Ophthalmology and imaging subspecialties as well as geographical regions of practice (Acknowledgment). Study recruitment was conducted using standardized invitations sent by the associations *via* their official established channels with all actively enrolled members. Recruitment was led by a study team member that was a member in good standing in each participating professional association.

The initial invitation to participate was sent to all actively enrolled members within each association. All invitations were sent by email and supplemented by regional practices based on the societies established channels with their members, such as WeChat in China. Invitations included the unique uniform resource locator (URL) of the web-based survey, that was programmed to restrict entries to one per participant-device to avoid duplication of entries from providers enrolled in multiple associations and/or receiving invites from multiple channels. Invitations were followed by 3 reminders at ~2-week intervals, coordinated by the study team members.

### Statistical Analysis

Responses were described with valid percentages for categorical variables as well as mean and standard deviation (SD) for continuous variables, with response rate tabulated for each question. The geographical origin of participants is classified based on the World Bank (WB) classification for 7 global regions (35). The economic background of participants is categorized using the 2017 International Council of Ophthalmology (ICO) classifications for low/intermediate and high resource settings, whereby countries grouped under resource-constrained settings were those classified by the WB as low- to upper-middle- income economies, and countries under resource-abundant settings were those classified by the WB as high-income economies (35, 36).

Quantitative analysis of any associations between provider acceptance and demographic information are reported. Multivariable logistic regression was performed to investigate any linear associations between provider acceptance of AI application in Ophthalmology and demographic information including age, gender, country (region of practice), economic background, experience, and self-rated understanding of AI for participants. To obtain a 95% confidence interval with 5% for the margin of error and 50% response distribution, a minimum sample size of 385 was calculated for the outcome of willingness to adopt AI in the next 5 years. Statistical significance was set at a *p*-value of 0.05. Analysis was performed using SPSS (IBM, SPSS Inc, USA).

In addition, machine learning (ML) analysis of survey responses was conducted using six selected input variables (clinical practice experience, World Bank geographical region, 2017 ICO classification for resource availability, gender, age, and self-reported AI understanding), to predict a total of 15 outcomes (output variables). An independent random forest model was trained to predict each outcome from the input variables in an exploratory analysis to assess for any non-linear associations between provider acceptance of AI and demographic information. The training dataset was randomly divided into 1,000 subjects for training, and 176 subjects for validation. To train each random forest model, five-fold cross-validation was first performed on the training dataset, to optimize four hyperparameters: the entropy criterion, the maximum depth of the random forest trees, the maximum number of features, and the number of tree estimators. The optimal hyperparameters thus found were then used on train the final model on the full training dataset, and subsequently applied to the validation dataset to evaluate the area under the receiver operating curve (AUC).

The Breiman-Cutler permutation importance measure was used to determine the most important input variable(s) in predicting each outcome (37). The permutation importance measure was computed by permuting the column values of a single input variable, and calculating the drop in overall accuracy caused by the permutation. For the outcome variables with six initial options (Strongly Agree, Agree, Neutral, Disagree, Strongly Disagree, Unsure), Strongly Agree and Agree were grouped together as positive outcomes, with the remaining options considered negative outcomes. For the outcome variables with three initial options (Yes, No, Unsure), Yes was considered a positive outcome, and No/Unsure as negative outcomes.

## Results

**Figure 1:**
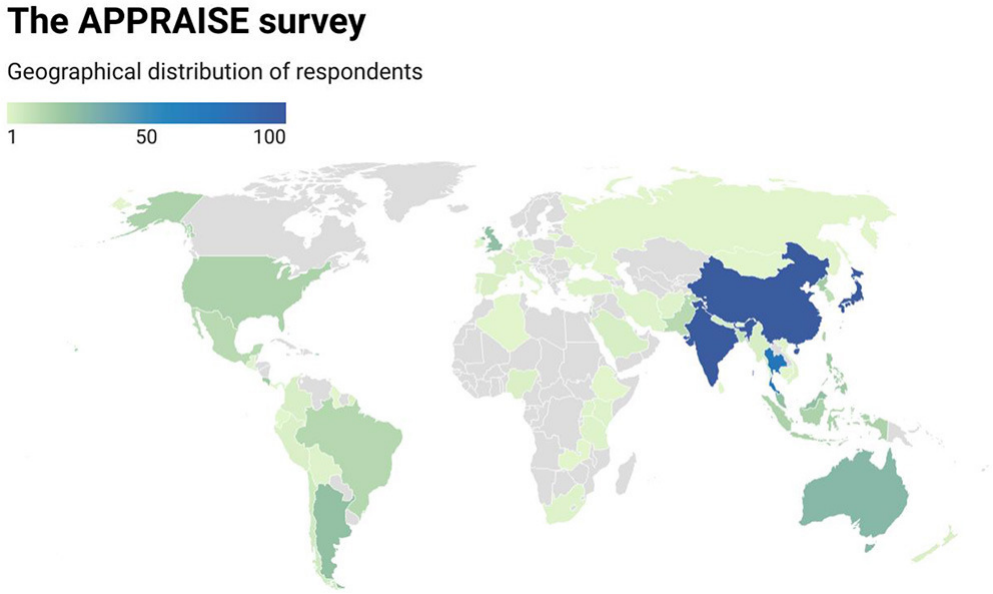
Geographical origin of participants to the APPRAISE survey.

A total of 1,176 ophthalmologists from 70 countries responded to the survey with representation from all 7 world bank geographical regions (Figure 1), although a majority practice in the East Asia & Pacific (74.0%), South Asia (10.9%) and Latin America & Caribbean (7.4%) regions. Participants had a mean age of 46.7 +/– 10.9 years. There was a slightly increased number of 597 male (50.8%) compared to 430 female (36.6%) participants, whereby 149 (12.7%) participants opted not to disclose their gender.

Participants reported a spectrum of clinical experience mostly between 10 and 30 years, whereby 323 participants had 10–20 years (27.5%) and 337 participants 20–30 years (28.7%) of experience. When asked to rate their understanding about machine learning (ML), deep learning (DL), and artificial intelligence (AI), a majority self-rated their understanding as average (54.3%, *n* = 639/1,176). Participant demographics are detailed in Table 1.

**Table 1:** Demographics of participants.

**Question**	**Responses**	***N*** **(%)**
Gender	Female	430 (36.6)
	Male	597 (50.8)
	Prefer not to say	149 (12.7)
Age (years)	Mean, Standard deviation (SD)	46.84, 10.936
Clinical practice experience in eye care services (eye screening, optometry, ophthalmology, etc)	Not practicing in eye care	13 (1.1)
	Currently in training (students in Ophthalmology and/or Optometry)	28 (2.4)
	< 5 years clinical practice experience	73 (6.2)
	5–10 years clinical practice experience	183 (15.6)
	10–20 years clinical practice experience	323 (27.5)
	20–30 years clinical practice experience	337 (28.7)
	>30 years clinical practice experience	219 (18.6)
Participant region of practice using world bank classification (All: Missing 2)	East Asia & Pacific	870 (74.0)
	Europe & Central Asia	51 (4.3)
	Latin America & the Caribbean	87 (7.4)
	Middle East & North Africa	20 (1.7)
	North America	13 (1.1)
	South Asia	128 (10.9)
	Sub-Saharan Africa	7 (0.6)
How would you rate your understanding about deep learning, machine learning, and AI?	Excellent	66 (5.6)
	Above average	226 (19.2)
	Average	639 (54.3)
	Below average	196 (16.7)
	Very poor	49 (4.2)

### Microsystem—Professional Acceptance of Clinical AI Applications for Eye Care

**Figure 2:**
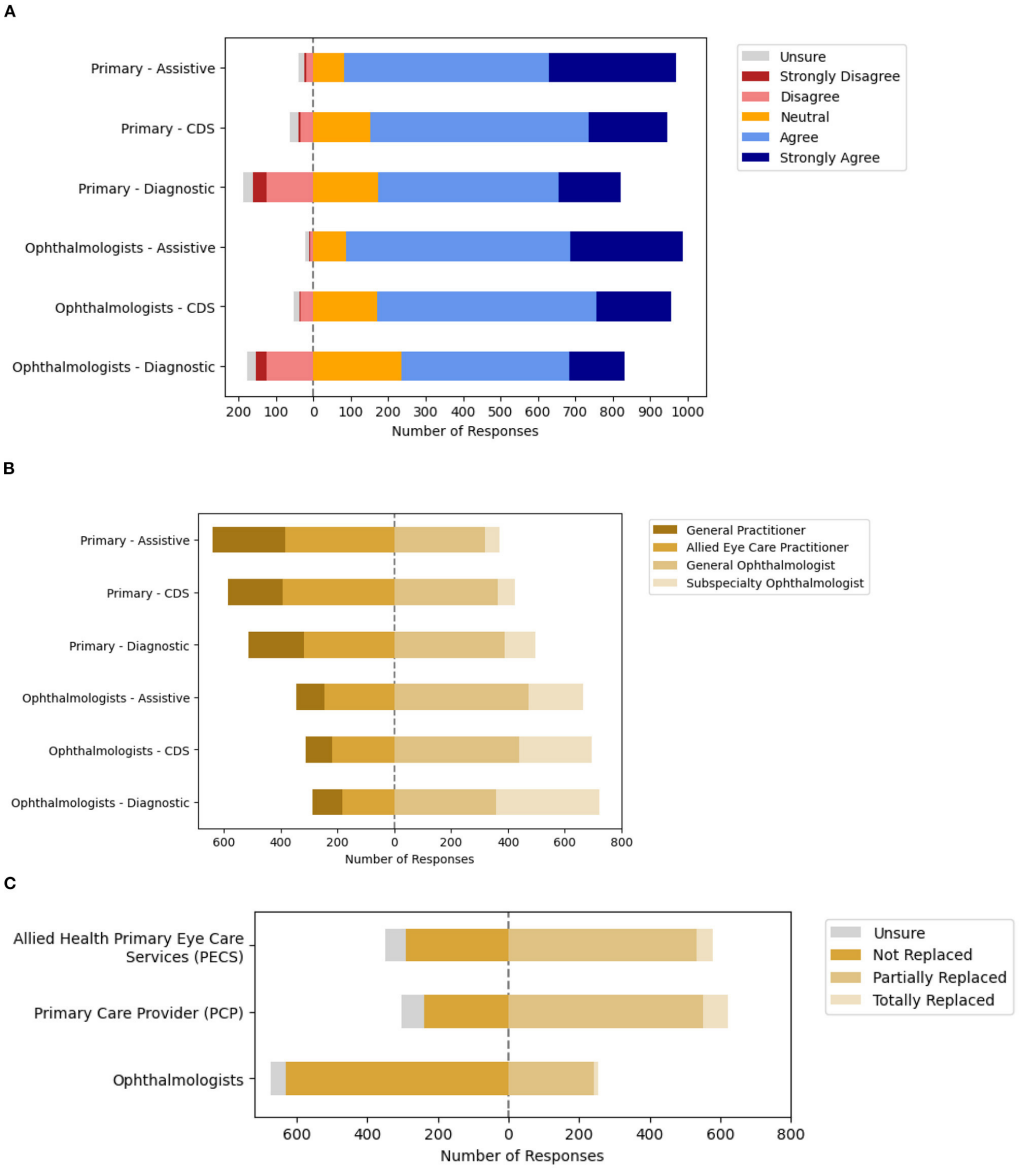
Ophthalmologists acceptance of artificial intelligence (AI) based on the software as a medical device (SaMD) regulatory framework. **(A)** Acceptance of clinical AI based on significance of information and intended user. **(B)** Acceptable level of error based on significance of information and intended user. **(C)** Anticipated impact of clinical AI on professional roles.

Participants were asked about their acceptance of various applications of AI for eye care services based on the solutions' intended user and clinical application in accordance with the SaMD regulatory framework. Assistive tools to inform clinical management were the most acceptable form of clinical AI application in ophthalmology, with applications designed for use by ophthalmologists (89.2%, *n* = 901/1,010) receiving higher acceptance than those intended for use by Primary Eye Care Providers (88.1%, *n* = 890/1,010). Professional acceptance of AI applications as CDS tools to drive clinical management received lower acceptance. Diagnostic tools intended for use by ophthalmologists received the lowest (59.1%, *n* = 597/1,010) acceptance among the 6 categories (Figure 2A, Supplementary Table 1A**)**.

Multivariate analysis was also conducted for the professional acceptance of clinical AI applications based on their intended users with demographic factors included for adjustment (Table 2). In this model, the odds of professional acceptance of AI applications for PECPs as assistive tools was lower among participants practicing in Latin America and the Caribbean (OR 0.42, 95% CI: 0.19–0.90, *p* = 0.025) than those practicing in East Asia and the Pacific. However, acceptance of AI applications for PECPs as diagnostic tools was relatively higher among participants that self-rated their understanding of AI as average (OR 1.06, 95% CI: 0.01–0.84, *p* = 0.033) or above average (OR 1.21, 95% CI: 0.02–0.95, *p* = 0.044).

**Table 2:** Multivariate analysis for professional acceptance of artificial intelligence (AI) applications in Ophthalmology.

		**Assistive tool for PECPs**				**CDS tool for PECPs**				**Diagnostic tool for PECPs**				**Assertive tool for ophthalmologists**				**CDS tool for ophthalmologists**				**Diagnostic tool for ophthalmologists**			
		**OR**	**95% CI**		***p*** **-value**	**OR**	**95% CI**		***p*** **-value**	**OR**	**95% CI**		***p*** **-value**	**OR**	**95% CI**		***p*** **-value**	**OR**	**95% CI**		***p*** **-value**	**OR**	**95% CI**		***p*** **-value**
Age		0.982	0.953	1.012	0.236	1.002	0.977	1.027	0.884	1.020	0.999	1.042	0.065	1.002	0.971	1.035	0.892	1.017	0.992	1.044	0.188	1.026	1.004	1.049	0.019
Gender	Female	Ref	Ref	Ref	Ref	Ref	Ref	Ref	Ref	Ref	Ref	Ref	Ref	Ref	Ref	Ref	Ref	Ref	Ref	Ref	Ref	Ref	Ref	Ref	Ref
	Male	1.253	0.794	1.976	0.332	1.191	0.830	1.711	0.343	1.089	0.798	1.486	0.590	1.316	0.817	2.121	0.259	1.434	0.991	2.076	0.056	1.398	1.020	1.914	0.037
Clinical experience	Currently in training	Ref	Ref	Ref	Ref	Ref	Ref	Ref	Ref	Ref	Ref	Ref	Ref	Ref	Ref	Ref	Ref	Ref	Ref	Ref	Ref	Ref	Ref	Ref	Ref
	<20 years	2.158	0.674	6.910	0.195	0.806	0.287	2.263	0.683	1.018	0.446	2.323	0.966	2.450	0.956	6.280	0.062	0.951	0.389	2.325	0.912	0.747	0.328	1.700	0.487
	>20 years	1.676	0.437	6.423	0.451	0.700	0.214	2.291	0.556	1.007	0.382	2.654	0.988	3.702	1.096	12.510	0.035	1.165	0.395	3.436	0.782	1.037	0.394	2.727	0.941
Geographical region	East Asia and Pacific	Ref	Ref	Ref	Ref	Ref	Ref	Ref	Ref	Ref	Ref	Ref	Ref	Ref	Ref	Ref	Ref	Ref	Ref	Ref	Ref	Ref	Ref	Ref	Ref
	Europe and Central Asia	1.200	0.450	3.204	0.716	1.000	0.475	2.103	0.999	1.334	0.700	2.543	0.380	0.638	0.274	1.486	0.298	1.292	0.574	2.908	0.536	1.068	0.565	2.018	0.840
	Latin America & the Caribbean	0.415	0.192	0.898	0.025	0.722	0.381	1.369	0.318	1.010	0.568	1.797	0.973	3.595	0.809	15.987	0.093	1.297	0.599	2.809	0.509	2.195	1.137	4.236	0.019
	Middle east and North Africa	0.578	0.153	2.187	0.419	0.832	0.254	2.721	0.761	0.833	0.290	2.393	0.735	1.793	0.224	14.323	0.582	0.996	0.266	3.728	0.995	0.740	0.250	2.188	0.586
	North America	1.000				2.965	0.372	23.658	0.305	1.869	0.488	7.160	0.361	1.174	0.145	9.542	0.881	0.983	0.205	4.703	0.983	2.210	0.567	8.615	0.253
	South Asia	0.874	0.381	2.006	0.751	1.008	0.552	1.840	0.980	0.987	0.595	1.637	0.960	0.598	0.287	1.249	0.171	0.785	0.432	1.428	0.428	0.604	0.364	1.000	0.050
	Sub-Saharan Africa	0.455	0.049	4.244	0.490	0.480	0.083	2.777	0.413	0.661	0.107	4.090	0.656	1.000				0.554	0.095	3.245	0.513	2.061	0.222	19.089	0.524
Income level	Resource-constrained	Ref	Ref	Ref	Ref	Ref	Ref	Ref	Ref	Ref	Ref	Ref	Ref	Ref	Ref	Ref	Ref	Ref	Ref	Ref	Ref	Ref	Ref	Ref	Ref
	Resource-abundant	0.630	0.345	1.150	0.132	0.914	0.583	1.434	0.696	0.738	0.501	1.086	0.124	0.776	0.422	1.428	0.416	0.799	0.502	1.270	0.342	0.543	0.367	0.805	0.002
Self-rated understanding of AI	Very poor	Ref	Ref	Ref	Ref	Ref	Ref	Ref	Ref	Ref	Ref	Ref	Ref	Ref	Ref	Ref	Ref	Ref	Ref	Ref	Ref	Ref	Ref	Ref	Ref
	Below average	0.713	0.078	6.553	0.765	1.286	0.315	5.261	0.726	0.418	0.050	3.489	0.420	0.611	0.070	5.293	0.655	1.058	0.295	3.798	0.931	0.851	0.210	3.450	0.821
	Average	0.587	0.073	4.714	0.616	0.840	0.226	3.125	0.795	0.107	0.014	0.837	0.033	0.608	0.076	4.865	0.639	1.499	0.441	5.094	0.517	0.325	0.086	1.226	0.097
	Above average	0.575	0.071	4.632	0.603	1.061	0.284	3.968	0.930	0.121	0.016	0.949	0.044	0.670	0.083	5.379	0.706	1.712	0.502	5.842	0.391	0.324	0.086	1.223	0.096
	Excellent	0.620	0.069	5.557	0.669	1.196	0.284	5.030	0.807	0.137	0.017	1.124	0.064	0.643	0.072	5.766	0.693	1.525	0.400	5.814	0.537	0.418	0.102	1.709	0.225

^*^Wherein “ref” denotes the reference category.

The color values are added to draw attention of readers to analyses for which p-value was < 0.05.

On the other hand, the odds of professional acceptance of AI applications for ophthalmologists as assistive tools was relatively higher among those with clinical experience of 20 or more years (OR 3.70, 95% CI: 1.10–12.5, *p* = 0.035). Similarly, acceptance of AI applications for ophthalmologists as diagnostic tools was relatively higher among participants with increasing age (OR 1.03, 95% CI: 1.00–1.05, *p* = 0.019), male gender (OR 1.40, 95% CI: 1.02–1.91, *p* = 0.037), and participants practicing in the Latin America and the Caribbean region (OR 2.20, 95% CI: 1.14–4.24, *p* = 0.019), although it was lower among resource-abundant practice settings (OR 1.36, 95% CI: 0.37–0.80, *p* = 0.002). No demographic variables had statistically significant associations with professional acceptance of AI applications as CDS tools for either group of intended users.

Next, participants reported the acceptable level of error for the various applications of AI for eye care services based on the intended user and application, when level of error was benchmarked against various practitioners (PECPs, general practitioners, general, and subspecialty-trained ophthalmologists). Overall, participants had greater expectations for the performance of clinical AI applications intended for use by ophthalmologists as opposed to that for use by PECPs (Figure 2B, Supplementary Table 1B). Specifically, the acceptable levels of error for Assistive and CDS applications of AI that were reported most frequently was the level of error equivalent to the intended user (whether PECP or ophthalmologist). On the other hand, the acceptable level of error for AI applications as diagnostic tools varied based on the intended user. The acceptable level of error for diagnostic tool applications of AI intended to be used by PECPs that was most frequently reported was a level of error equivalent to a general ophthalmologist (38.5%, *n* = 389/1,010). Participants were divided about the acceptable level of error for diagnostic tool applications of AI intended to be used by ophthalmologists, with equivalent to a general ophthalmologist (35.5%, *n* = 359/1,010) or subspecialty-trained ophthalmologist (36.0%, *n* = 364/1,010) being the most frequent responses.

Next, participants were surveyed about the potential impact of clinical AI on their professional roles and responsibilities at the level of the healthcare microsystem. The majority of participants that responded indicated that the eye care roles of ophthalmologists are not likely to be replaced (68.2%, *n* = 632/927), although those of others may be partially replaced including allied primary eye care service (PECS) providers (57.6%, *n* = 534/927) and primary care providers (PCP) with eye care services (59.3%, *n* = 553/927). Detailed responses are included in Figure 2C and Supplementary Table 1C.

**Figure 3:**
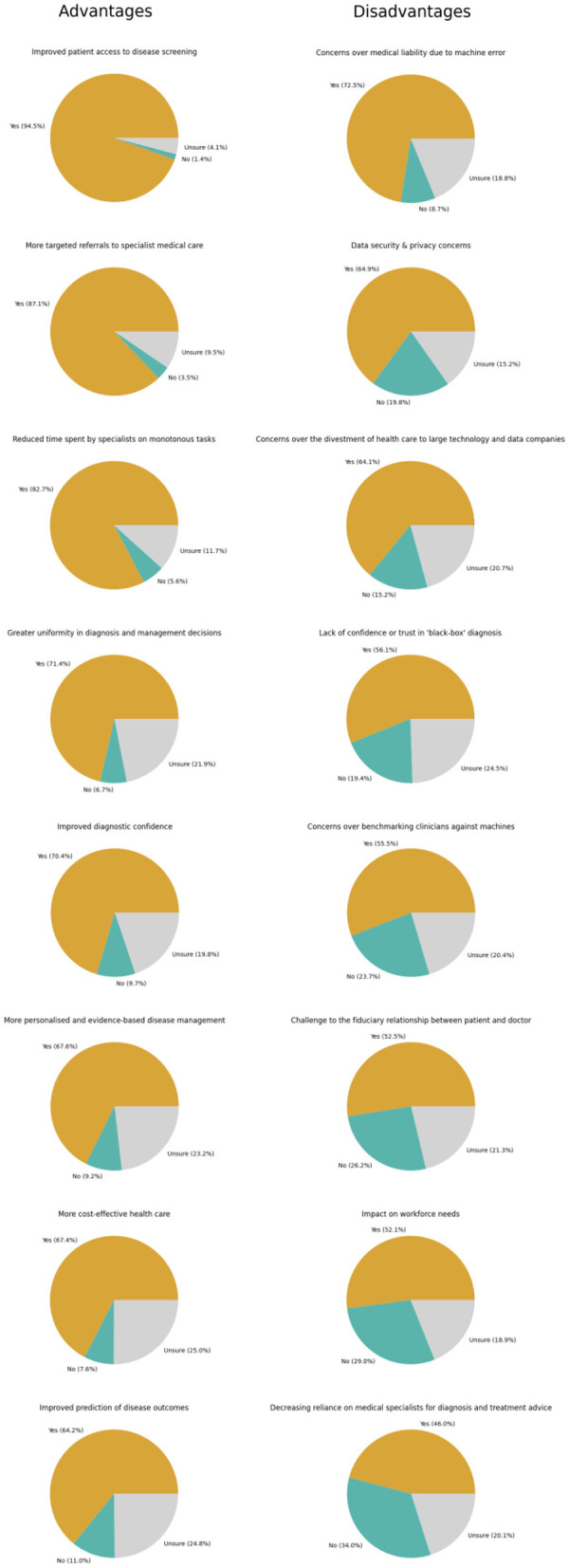
Perceptions regarding advantages and disadvantages of artificial intelligence (AI) of ophthalmologists.

Finally, participants were surveyed about their perceptions of potential advantages and disadvantages of clinical AI for Ophthalmology to identify potential barriers and enablers for clinical AI adoption. Overall, the perceived advantages of clinical AI for Ophthalmology that were most frequently reported include improved patient access to disease screening (94.5%, *n* = 876/927), more targeted referrals to specialist care (87.1%, *n* = 807/927), and reduced time spent by specialists on monotonous tasks (82.7%, *n* = 767/927). The disadvantages of clinical AI for Ophthalmology that were most frequently reported include concerns over medical liability due to machine error (72.5%, *n* = 672/927), data security & privacy concerns (64.9%, *n* = 602/927), and concerns over the divestment of healthcare to large technology and data companies (64.1%, *n* = 594/927). Further detailed responses are depicted in Figure 3 and Supplementary Tables 1D,E.

### Mesosystem—Organizational Adoption of AI for Clinical and Eye Care Services

Participants were asked about the willingness to adopt clinical artificial intelligence (AI) in their organizations. Six hundred four participants (51.4%) reported that their organizations were willing to adopt AI for clinical practice in general within the next 5 years. A multivariate logistic regression model was applied to evaluate associations with participant demographics.

In this model, the odds of participants indicating organizational willingness to adopt AI within 5 years was higher among ophthalmologists of male gender (OR 1.58, 95% CI: 1.18–2.10) and those practicing in the North American region (OR 8.54, 95% CI: 1.86–39.5, *p* = 0.006) compared to the East Asia and Pacific region. However, the odds of organizational willingness to adopt AI was lower among participants from resource-abundant regions (OR 0.39, 95% CI: 0.27–0.56, *p* < 0.001). There were no significant associations between the odds of organizational willingness to adopt AI and the remaining demographic factors, including age, clinical experience, and self-rated understanding of AI (Table 3A).

**Table 3A:** Organizational willingness to adopt AI for general eye practice.

		**Odds ratio**	**95% confidence interval**	***P*** **-value**
Age		1.007923	0.9889255	1.027285	0.416
Gender	Female	Ref	Ref	Ref	Ref
	Male	1.575258	1.181954	2.099436	0.002
Years of clinical experience	Student	Ref	Ref	Ref	Ref
	< 20 years	0.7718551	0.3476733	1.713564	0.525
	20 or more years	0.7120689	0.2867984	1.767939	0.464
Geographical region	East Asia and Pacific	Ref	Ref	Ref	Ref
	Europe and Central Asia	1.471252	0.8191418	2.642501	0.196
	Latin America & the Caribbean	1.220085	0.7132367	2.087116	0.468
	Middle east and North Africa	0.6701729	0.252713	1.77724	0.421
	North America	8.540024	1.84594	39.50941	0.006
	South Asia	1.586754	0.9715878	2.591415	0.065
	Sub-Saharan Africa	0.4363696	0.0930749	2.045863	0.293
Income level	Resource-constrained	Ref	Ref	Ref	Ref
	Resource-abundant	0.390912	0.2737758	0.5581654	<0.001
Participant self-rated understanding of artificial intelligence (AI)	Very Poor	Ref	Ref	Ref	Ref
	Below Average	1.105834	0.3658182	3.342835	0.859
	Average	0.8993603	0.3182563	2.541501	0.841
	Above Average	0.6707563	0.2368241	1.899781	0.452
	Excellent	0.7322512	0.2382404	2.250634	0.586

*Wherein “ref” denotes the reference category.

The color values are added to draw attention of readers to analyses for which p-value was < 0.05.

**Figure 4:**
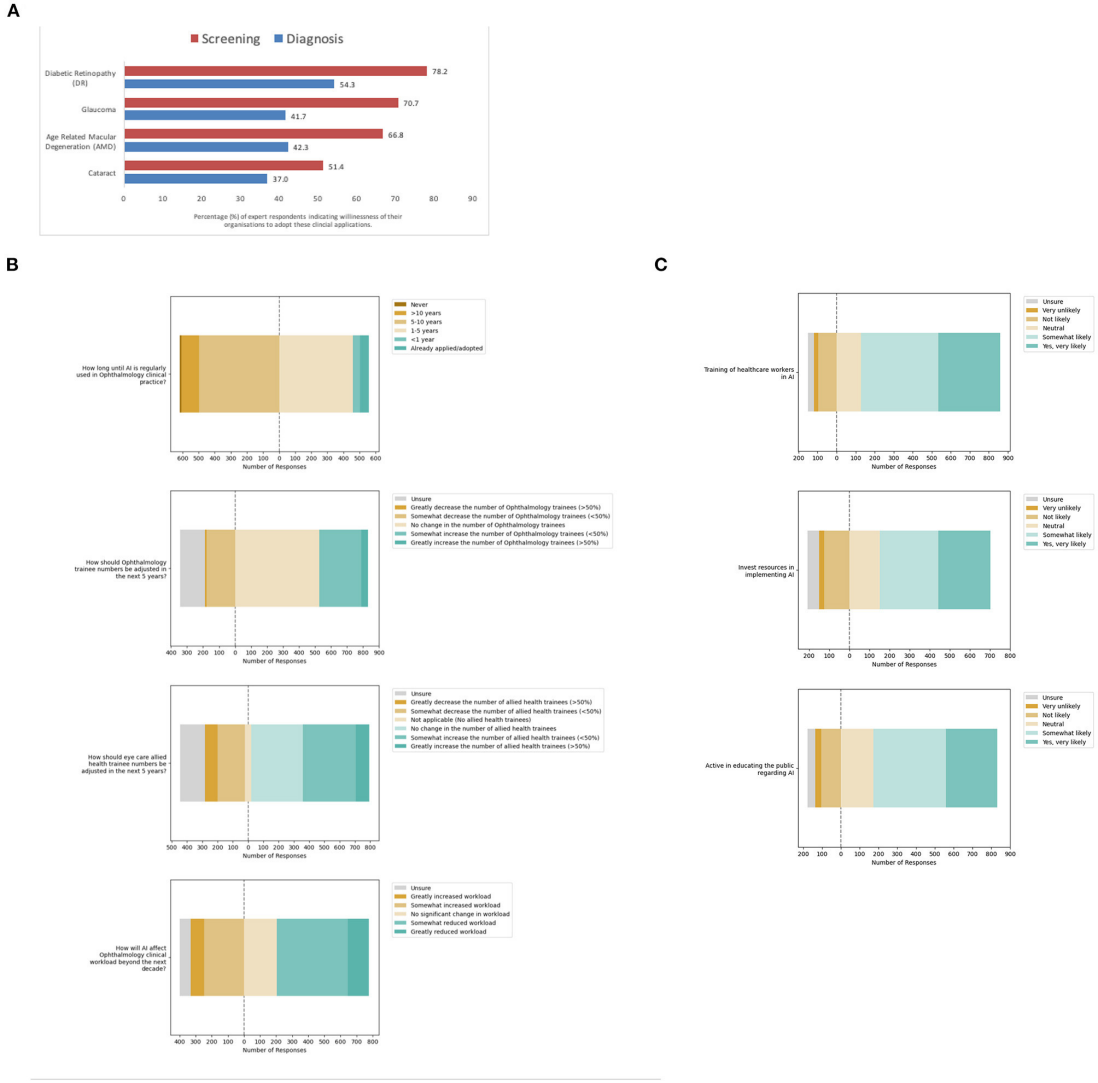
Meso-system—Organizational adoption of clinical artificial intelligence (AI). **(A)** Organizational willingness to adopt AI for specific eye care services. **(B)** Anticipated organizational impact from adoption. **(C)** Perceived likelihood of organizational facilitation of adoption.

Next, participants were asked about the perceived willingness of their organizations to adopt AI for leading validated applications in eye care services (Figure 4A). For screening applications of AI, most participants indicated organizational willingness to adopt AI including 920 participants for DR screening (78.2%), 832 for glaucoma screening (70.7%), 786 for AMD screening (66.8%), and 604 for cataract screening (51.4%). A multivariate logistic regression model was applied to evaluate associations between willingness to adopt AI for screening applications reported by participants, with their demographic factors (Table 3B).

**Table 3B:** Organizational willingness to adopt applications of AI in Screening for eye diseases.

		**Diabetic retinopathy (DR)**				**Glaucoma**				**Age related macular degeneration (AMD)**				**Cataract**			
		**OR**	**95% CI**		***p*** **-value**	**OR**	**95% CI**		***p*** **-value**	**OR**	**95% CI**		***p*** **-value**	**OR**	**95% CI**		***p*** **-value**
Age		1.023	0.999	1.047	0.056	1.020	0.999	1.041	0.066	1.018	0.998	1.039	0.075	1.021	1.002	1.041	0.03
Gender	Female	Ref	Ref	Ref	Ref	Ref	Ref	Ref	Ref	Ref	Ref	Ref	Ref	Ref	Ref	Ref	Ref
	Male	1.080	0.765	1.525	0.66	1.015	0.743	1.387	0.923	1.023	0.759	1.378	0.883	0.667	0.505	0.882	0.004
Clinical experience	Currently in training	Ref	Ref	Ref	Ref	Ref	Ref	Ref	Ref	Ref	Ref	Ref	Ref	Ref	Ref	Ref	Ref
	<20 years	1.237	0.511	2.998	0.637	1.150	0.519	2.550	0.73	1.068	0.485	2.351	0.871	0.724	0.329	1.593	0.422
	>20 years	0.972	0.349	2.709	0.957	1.172	0.463	2.971	0.737	1.047	0.419	2.613	0.922	0.748	0.303	1.846	0.529
Geographical region	East Asia and Pacific	Ref	Ref	Ref	Ref	Ref	Ref	Ref	Ref	Ref	Ref	Ref	Ref	Ref	Ref	Ref	Ref
	Europe and Central Asia	1.669	0.757	3.676	0.204	0.559	0.306	1.021	0.058	1.495	0.758	2.948	0.246	0.444	0.236	0.836	0.012
	Latin America & the Caribbean	1.631	0.767	3.470	0.204	1.695	0.899	3.195	0.103	1.691	0.931	3.073	0.085	0.863	0.515	1.446	0.576
	Middle east and North Africa	0.515	0.188	1.413	0.198	0.365	0.144	0.922	0.033	0.570	0.224	1.450	0.238	0.644	0.254	1.635	0.355
	North America	1.471	0.314	6.889	0.624	0.333	0.106	1.046	0.06	2.095	0.451	9.736	0.346	0.174	0.037	0.812	0.026
	South Asia	2.068	1.037	4.125	0.039	1.719	0.998	2.960	0.051	1.207	0.737	1.976	0.455	1.249	0.784	1.992	0.35
	Africa	1.180	0.137	10.176	0.88	2.248	0.263	19.214	0.459	0.373	0.080	1.732	0.208	1.597	0.296	8.615	0.586
Income level	Resource-constrained	Ref	Ref	Ref	Ref	Ref	Ref	Ref	Ref	Ref	Ref	Ref	Ref	Ref	Ref	Ref	Ref
	Resource-abundant	0.729	0.472	1.126	0.154	1.205	0.821	1.769	0.34	1.193	0.825	1.726	0.349	0.755	0.533	1.069	0.113
Self-rated understanding of AI	Very poor	ref	ref	ref	ref	ref	ref	ref	ref	ref	ref	ref	ref	ref	ref	ref	ref
	Below average	0.927	0.235	3.659	0.914	1.201	0.371	3.887	0.759	0.842	0.263	2.694	0.772	1.778	0.594	5.317	0.303
	Average	1.151	0.313	4.240	0.832	1.274	0.422	3.846	0.668	1.038	0.344	3.130	0.948	1.224	0.442	3.388	0.698
	Above average	0.859	0.234	3.156	0.818	1.090	0.361	3.295	0.878	0.855	0.283	2.582	0.782	1.126	0.406	3.123	0.82
	Excellent	0.539	0.137	2.127	0.378	0.658	0.202	2.141	0.486	0.718	0.220	2.340	0.583	1.129	0.376	3.389	0.828

*Wherein “ref” denotes the reference category.

The color values are added to draw attention of readers to analyses for which p-value was < 0.05.

In this model, the odds of organizational willingness to adopt AI being reported by ophthalmologists practicing in South Asia was relatively higher for DR screening (OR 2.07, 95% CI: 1.04–4.13, *p* = 0.039) compared to East Asia and the Pacific. On the other hand, that for glaucoma screening reported by ophthalmologists in the Middle east and North Africa was relatively lower for glaucoma screening (OR 0.365, 95% CI: 1.04–4.13, *p* = 0.033) compared to East Asia and the Pacific. Finally, the odds of organizational willingness to adopt AI for cataract screening being reported by ophthalmologists of older age was higher (OR 1.02, 95% CI: 1.00–1.04, *p* = 0.030), while that by ophthalmologists of male gender was lower (OR 0.67, 95% CI: 0.50–0.88, *p* = 0.04). That for cataract screening was similarly lower among those practicing in the Europe and Central Asia region (OR 0.44, 95% CI: 0.24–0.84, *p* = 0.012) and North American region (OR 0.174, 95% CI: 0.04–0.81, *p* = 0.026).

Notably, the perception of organizational willingness to adopt diagnostic applications of AI was lower than screening applications (Figure 4A). Fewer participants indicated organizational willingness to adopt AI for diagnostic applications: positive responses were recorded from 638 participants for DR (54.3%), 490 for glaucoma (41.7%), 497 for AMD (42.3%) and 435 for cataract diagnosis (37.0%). A multivariate logistic regression model was applied to evaluate associations between willingness to adopt AI for diagnostic applications reported by participants, with their demographic factors (Table 3C).

**Table 3C:** Organizational willingness to adopt applications of AI for Diagnosis of eye diseases.

		**Diabetic retinopathy (DR)**				**Glaucoma**				**Age related macular degeneration (AMD)**				**Cataract**			
		**OR**	**95% CI**	***p*** **-value**	**OR**	**95% CI**	***p*** **-value**	**OR**	**95% CI**	***p*** **-value**	**OR**	**95% CI**	***p*** **-value**
Age		1.023	1.004	1.042	0.02	1.036	1.016	1.056	0	1.021	1.002	1.041	0.029	1.018	0.998	1.038	0.072
Gender	Female	Ref	Ref	Ref	Ref	Ref	Ref	Ref	Ref	Ref	Ref	Ref	Ref	Ref	Ref	Ref	Ref
	Male	1.158	0.877	1.529	0.301	1.527	1.148	2.031	0.004	1.403	1.055	1.865	0.02	0.828	0.619	1.107	0.202
Clinical experience	Currently in training	Ref	Ref	Ref	Ref	Ref	Ref	Ref	Ref	Ref	Ref	Ref	Ref	Ref	Ref	Ref	Ref
	<20 years	1.369	0.628	2.982	0.429	1.143	0.493	2.651	0.755	1.667	0.705	3.944	0.245	0.771	0.343	1.732	0.528
	>20 years	1.221	0.501	2.976	0.66	0.860	0.334	2.212	0.754	1.904	0.729	4.972	0.188	0.936	0.372	2.357	0.888
Geographical region	East Asia and Pacific	Ref	Ref	Ref	Ref	Ref	Ref	Ref	Ref	Ref	Ref	Ref	Ref	Ref	Ref	Ref	Ref
	Europe and Central Asia	1.245	0.693	2.234	0.464	0.968	0.528	1.775	0.916	1.656	0.915	2.996	0.095	0.486	0.237	0.997	0.049
	Latin America & the Caribbean	1.801	1.043	3.109	0.035	1.242	0.735	2.098	0.418	1.829	1.077	3.106	0.026	0.737	0.427	1.272	0.273
	Middle east and North Africa	0.721	0.288	1.806	0.485	0.306	0.099	0.948	0.04	0.530	0.185	1.521	0.238	0.347	0.099	1.221	0.099
	North America	0.734	0.237	2.277	0.593	0.633	0.196	2.046	0.445	0.809	0.252	2.594	0.722	0.307	0.066	1.433	0.133
	South Asia	1.688	1.046	2.724	0.032	1.102	0.687	1.766	0.687	1.295	0.810	2.071	0.28	1.118	0.697	1.794	0.643
	Africa	1.733	0.324	9.272	0.521	2.734	0.504	14.826	0.243	0.453	0.084	2.454	0.358	3.202	0.594	17.247	0.176
Income level	Resource-constrained	Ref	Ref	Ref	Ref	Ref	Ref	Ref	Ref	Ref	Ref	Ref	Ref	Ref	Ref	Ref	Ref
	Resource-abundant	0.772	0.545	1.095	0.147	0.864	0.604	1.236	0.423	0.712	0.498	1.018	0.063	0.723	0.502	1.040	0.08
Self-rated understanding of AI	Very poor	Ref	Ref	Ref	Ref	Ref	Ref	Ref	Ref	Ref	Ref	Ref	Ref	Ref	Ref	Ref	Ref
	Below average	0.669	0.210	2.129	0.497	0.924	0.307	2.781	0.889	0.706	0.233	2.139	0.538	1.507	0.494	4.598	0.471
	Average	0.535	0.179	1.600	0.263	0.637	0.226	1.793	0.393	0.508	0.179	1.442	0.203	0.985	0.344	2.818	0.978
	Above average	0.436	0.145	1.305	0.138	0.573	0.203	1.618	0.293	0.475	0.167	1.351	0.163	1.028	0.359	2.948	0.959
	Excellent	0.628	0.194	2.030	0.437	0.656	0.215	2.001	0.458	0.755	0.246	2.322	0.624	1.214	0.391	3.763	0.737

*Wherein “ref” denotes the reference category.

The color values are added to draw attention of readers to analyses for which p-value was < 0.05.

In this model, the odds of organizational willingness to adopt AI being reported for DR diagnosis was higher for participants of older age (OR 1.02, 95% CI: 1.00–1.04, *p* = 0.020). That for DR diagnosis was also higher among participants practicing in the Latin America & the Caribbean region (OR 1.80, 95% CI: 1.04–3.11, *p* = 0.035) as well as the South Asian region (OR 1.69, 95% CI: 1.05–2.72, *p* = 0.032) relative to the East Asia and Pacific region. Similarly, the odds of organizational willingness to adopt AI being reported for Glaucoma diagnosis was higher for participants of older age (OR 1.04, 95% CI: 1.02–1.06, *p* < 0.001) and male gender (OR 1.53, 95% CI: 1.15–2.03, *p* = 0.004). However, that for Glaucoma diagnosis was lower for participants in the Middle east and North African region (OR 0.31, 95% CI: 0.10–0.95, *p* = 0.040).

Furthermore, the odds of organizational willingness to adopt AI being reported for AMD diagnosis was higher for participants of older age (OR 1.02, 95% CI: 1.00–1.04, *p* = 0.029) and male gender (OR 1.40, 95% CI: 1.06–1.87, *p* = 0.020). In addition, that for AMD diagnosis was also higher for participants practicing in the Latin America and the Caribbean region (OR 1.83, 95% CI: 1.08–3.11, *p* = 0.026) compared to the East Asia and the Pacific. On the other hand, the odds of organizational willingness to adopt AI being reported for Cataract screening were lower for participants practicing in the Europe and Central Asia region (OR 0.486, 95% CI: 0.24–1.00, *p* = 0.049) compared to the East Asia and the Pacific region.

Next, participants were asked about the anticipated organizational impact of the adoption of clinical AI for eye care services (Figure 4B, Supplemental Table 2A). Interestingly, some 55 participants indicated AI was already adopted for eye care services in their organizations (4.7%). Most of these participants had self-rated their understanding of clinical AI as excellent (16.4%, 9/55) or above average (27.3%, 15/55). These included 17 of the participants from the South Asian region (13.3%), 2 of the participants from Europe and central Asia region (3.9%), 33 of the participants from the East Asia and Pacific region (3.8%), and 3 of the participants from the Latin America and the Caribbean region (3.4%).

Despite the current progress in validation and implementation of AI for eye care services, less than half of all participants in this survey felt that AI would be regularly used in clinical practice within the next 5 years (47.4%, *n* = 558/1,176). Furthermore, participants had mixed views regarding the impact of AI on ophthalmology clinical workload, with some anticipating reduced workload (48.6%, *n* = 572/1,176) and others instead anticipating increased workload (28.2%, *n* = 332/1,176). When asked if trainee numbers should be increased, decreased or kept the same, most participants indicated that ophthalmology trainee numbers should not be adjusted (44.8%, *n* = 527/1,176), although some indicated allied health eye care trainee numbers should be increased (37.0%, *n* = 435/1,176).

With respect to their organizational willingness to facilitate adoption of AI tools for eye care services, study participants were optimistic overall. Many participants indicated that their organizations were very likely or somewhat likely to specifically train healthcare workers in the use and understanding of AI (72.3%, *n* = 730/1,010), invest resources for implementation (54.5%, *n* = 550/1,010), and actively educate the public regarding the use of AI in Ophthalmology (65.3%, *n* = 660/1,010). Detailed responses are included in Figure 4C and Supplemental Table 2B.

### Macrosystem—Value of Clinical AI Applications for Eye Care Across the Health System

**Figure 5:**
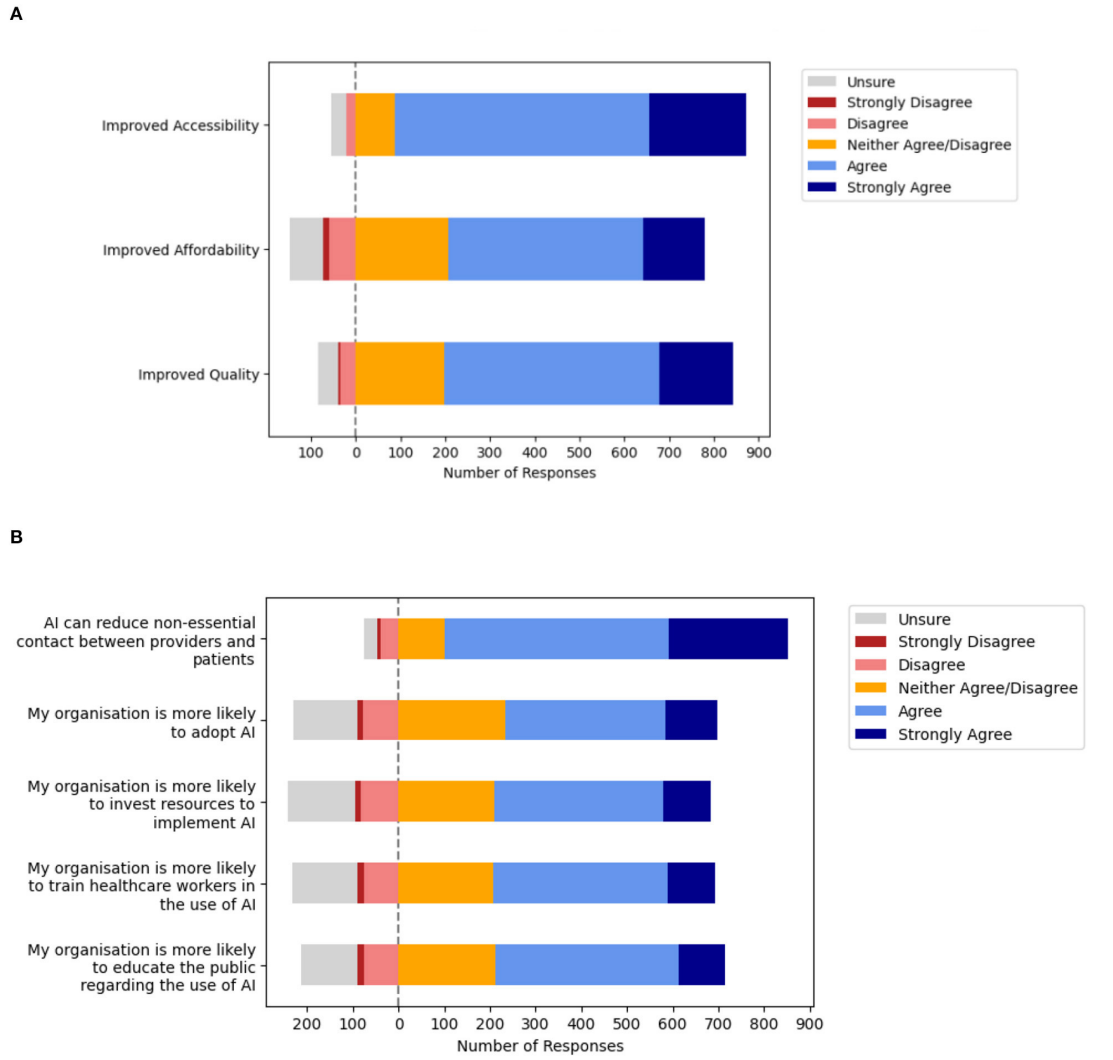
Macrosystem—Ophthalmologists perceptions about artificial intelligence (AI) and the impact of the pandemic on adoption within health systems. **(A)** Value of clinical artificial intelligence (AI) perceived by Ophthalmologists. **(B)** Impact of the coronavirus disease 2019 (COVID-19) on health system adoption.

Many participants indicated that they strongly agree or agree that clinical AI will facilitate improvements in accessibility (84.7%, *n* = 785/927), affordability (61.9%, *n* = 574/927), and quality (69.4%, *n* = 643/927) in eye care services. Detailed responses are depicted in Figure 5A and Supplemental Table 3A. Next, participants were surveyed about their perceptions regarding the impact of COVID-19 acting at the level of the healthcare macrosystem (Figure 5B, Supplemental Table 3B). Notably, many participants were optimistic regarding the potential for AI to reduce non-essential contact between providers and patients (80.9%, *n* = 750/927). However, participants were closely divided regarding whether COVID-19 increased the likelihood of organizational AI adoption (50.2%, *n* = 465/927) as well as organizational facilitation. Participants remained divided when the likelihood of organizational facilitation was explored in greater detail in terms of investing resources to implement AI (51.1%, *n* = 474/927), training healthcare workers in AI (52.4%, *n* = 486/927), and educating the public regarding AI (54.2%, *n* = 502/927).

### Machine Learning Analysis for Clustering of Survey Responses

On analysis of the survey responses using machine learning (ML) models, predictive AUCs of between 0.52 and 0.83 were obtained in predicting binary outcomes with corresponding permutation importance depicted in Appendix 2. The outcome variable predicted with the highest AUC of 0.83 was on whether AI could be an acceptable assistive tool for ophthalmologists, whereas the AUC for predicting the application of AI as a diagnostic tool for ophthalmologists had relatively low values of 0.59 or below. Finally, the model achieved an AUC of 0.65 in predicting organizational willingness to adopt AI in clinical practice in 5 years, whereby the variables that had the greatest predictive value were those for self-reported AI understanding and resource availability, with clinical practice experience having low predictive value. Detailed results are demonstrated in the Appendix 2.

## Discussion

To our knowledge, this is the first study providing an in-depth evaluation of ophthalmologists acceptance of clinical AI for Ophthalmology that incorporates the relevant medical device regulatory framework. Provider perspectives on professional and organizational acceptance of clinical AI tools for eye care services are evaluated in this study involving participants from a spectrum of geographies and clinical subspecialties. A machine learning (ML) approach was applied to highlight the clustering of responses, illustrating the relevance of individual demographic and attitude variables on professional acceptance and likelihood of adoption. Overall, participants indicated high levels of professional and organizational acceptance of AI for eye care services. Potential important barriers and enablers for the implementation of these tools in clinical practice were also highlighted. Furthermore, the impact of COVID-19 on clinical AI adoption in Ophthalmology was assessed.

### Healthcare Micro-System Considerations for the Implementation of Clinical AI

The results of this study suggest several considerations for facilitating adoption of clinical AI at the level of healthcare microsystems. Participants were more accepting of clinical AI applications as assistive tools rather than CDS or diagnostic tools, based on the software as a medical device (SaMD) regulatory framework for clinical intended uses of AI technology to inform clinical management by highlighting areas of interest, drive clinical management by initiating referrals, or diagnose eye diseases to recommend management, respectively. The process of forming a clinical diagnosis is a fundamental role of healthcare practitioners. It is a complexed art based on probabilistic, causal or deterministic reasoning, often without the availability of complete information (38). The practitioner has to identify patterns in clinical information about each individual patient in the context of their prior medical and contextual knowledge to form an impression, then validate it through trial of treatment or investigations (39). This may explain why assistive tools received the greatest acceptance as opposed to CDS or diagnostic tools that suggest or provide a diagnosis, given the inability to incorporate additional contextual and non-verbal information in AI for a holistic approach to evaluating patients (Figure 2A).

The perceived enablers of improved accessibility and optimized referrals from screening as well as acceptance of these tools when designed for used by allied PECPs also suggests avenues to optimize solution development and deployment. Applying design considerations to facilitate operationalising clinical AI used by PECPs within the community to filter out patients with advanced illness requiring tertiary care are more acceptable to stakeholders. This may require embedded systems to facilitate referrals to ophthalmologists using the sorting conveyor or pyramid operational models where required (31).

Most ophthalmologists were not concerned about the threat of being replaced by AI (Figure 2C). The views of participants in this study are consistent with that from studies in other fields including Pathology and Radiology, 2 other medical fields with leading applications of AI for classification of medical imaging (11, 40). Many Diagnostic Pathologists in recent study reported that a negative impact of clinical AI on their professional compensation was unlikely (65.6%) and displacement or negative career impacts were limited (38.0%). They instead anticipated an increase in employment prospects (42.4%) (28). Similarly, among European Radiologist participants, most anticipated an increase in job opportunities (58%), with increased clinical roles (54%) and decreased administrative roles such as reporting (75%) (27), in keeping with the advantage of reduced monotonous tasks perceived by participants.

Therefore, we find that participants are confident in their clinical roles and do not perceive AI to be a major threat to professional roles. This is consistent with the lower agreement reported for relevant potential disadvantages of AI (Figure 3), such as decreased reliance of medical specialists for diagnosis and treatment advice. These results are also consistent with an acceptance survey conducted across 22 provinces in China, whereby few healthcare workers anticipate replacement of clinical activities with AI (6.0%), while being receptive to applications that assist diagnosis (40.0%) and treatment (39.2%) (41).

The major disadvantages of clinical AI that participants agreed upon include potential medical liability from machine error, data security, privacy, and potential divestment of healthcare to corporate entities (Figure 3). Yet, despite these limitations, it has been successfully trained and validated for classification tasks of medical imaging for screening and diagnosis with clinically acceptable performance (42). The progress in this technology is reflected in the high rates of provider acceptance for the various abovementioned clinical applications. Furthermore, participants largely agreed on the advantages of AI (Figure 3), including improved patient access to disease screening, targeted referrals to specialist medical care, and reduce time spent on monotonous tasks. Decentralized and improved access to screening has increased relevance today given widespread fear of viral exposure within hospitals that has prompted many patients to post-pone regular eye screening and monitoring (43, 44). However, fewer participants agreed that AI would improve care by making it more personalized, cost-effective, or predictive to pre-empt the clinical needs of patients.

These findings highlight the need for greater stakeholder engagement to emphasize advantages of AI in tandem with research to address disadvantages perceived by experts (31, 45). For example, participants flagged up lack of trust and confidence in the “black-box” diagnosis inherent with existing solutions, which could be addressed by emerging solutions such as saliency maps to improve algorithmic transparency. More pragmatic and qualitative investigations of AI implementation to address these potential barriers and enablers of adoption are needed to facilitate successful implementation of clinical AI in practice (46).

### Healthcare Meso-System Considerations for the Implementation of Clinical AI

The analysis of healthcare meso-system considerations for clinical AI implementation highlight several trends in receptiveness to the adoption of clinical AI at the level of specific eye care services and within healthcare organizations. Overall, participants reported greater likelihood of organizational willingness to adopt screening applications rather than diagnostic applications. There was greater acceptance of applications for detection of DR, particularly in South Asia, and lower acceptance of applications for detection of Cataract, particularly in Europe and Central Asia (Tables 3B,C). This may relate to the importance of symptoms in the clinical evaluation of Cataracts, whereby screening models that incorporate AI screening or diagnostic applications within telemedicine platforms may facilitate real-world operational adoption (47).

Interestingly, the odds of reporting organizational willingness to adopt AI for certain applications were higher among participants with advanced age, including applications for detection of DR, glaucoma and AMD, as well as screening for cataracts (Tables 3B,C). This is congruent with the results of prior studies that have suggested increasing age may not be negatively correlated with health technology acceptance (41, 48). Furthermore, the odds of reporting organizational willingness to adopt AI were higher among participants with male gender for detection of glaucoma and AMD, although they were lower for cataract. These findings for AI adoption reflect the facilitating conditions, subjective norm, and social influence factors required for successful technology adoption from established theoretical models such as the technology acceptance model (TAM) and unified theory of acceptance and use of technology (UTAUT) (49, 50).

Yet, despite all the progress in the field of AI for ophthalmology, less than half of participants felt that AI is likely to be implemented in the next 5 years nor likely to reduce clinical workload (Figure 4). It follows that participants felt ophthalmology trainee numbers should not be adjusted (44.8%, *n* = 527/1,176). In ophthalmology, confidence in professional responsibilities likely stems from the procedural and surgical roles of professionals that cannot be replaced by AI. This interventional workload will likely increase with enhanced detection of eye diseases through the use of clinical AI to scale-up screening services, as reflected in the advantages of clinical AI anticipated by participants (Figure 3) including improved patient access to eye screening (94.5%) and targeted referrals to specialists (87.1%).

Furthermore, current evidence supports the improved cost-effectiveness of AI for eye care when applied in semi-autonomous models due to lower false positive referrals (17), highlighting that AI applied in partnership with healthcare practitioners will likely result in superior outcomes. In addition, apart from interventions, provision of clinical care also requires considerable management of technology for operational and administrative requirements. Earlier studies have highlighted that implementation of new technology such as electronic medical records (EMRs) can lead to delays and reduced efficiency in eye care services (51), requiring added time to review and interpret information (52). This highlights the need for design thinking approaches in the development of these tools, to streamline the aggregation and visualization of clinically relevant information from AI that can be conveniently interpreted by practitioners and applied in clinical practice (30).

Notably, most participants reported that their organizations are currently likely to facilitate adoption (Figure 4) through training healthcare workers in AI, investing resources for implementation, and actively educating the public. Based on the disadvantages reported in this study (Figure 3), remaining barriers that need to be addressed for adoption include potential medical liability arising from machine error (72.5%), data security & privacy (64.9%), as well as potential divestment of healthcare to large technology and data companies (64.1%). These can be addressed through the engagement of relevant stakeholders to develop medicolegal and cybersecurity guidelines, as well as co-development of these tools with the concerted involvement of relevant clinical participants (7, 16). Enablers to facilitate adoption include improving access to eye screening (94.5%), optimizing the flow of patients within eye care services for more targeted referrals to specialists (87.1%), and reducing the need for specialists to spend time on monotonous tasks (82.7%), that can be targeted as operational outcomes or goals during health services research and product development.

### Health Macro-System Considerations and Impact of COVID-19 on Clinical AI Implementation

Finally, factors affecting AI adoption at the level of the healthcare macrosystem were evaluated including perceptions regarding value of clinical AI and the impact of the pandemic on the likelihood of adoption. Participants were generally positive regarding the value of clinical AI. Most agreed that clinical AI for eye care services will improve the accessibility of eye care services (84.7%), although they were less certain regarding improvements in affordability (61.9%) and quality (69.4%). Moreover, having experienced the macrosystem changes brought about by the coronavirus disease 2019 (COVID-19) pandemic, participants were optimistic about the potential value of clinical AI to minimize non-essential patient contact (80.9%). However, participants were divided regarding whether COVID-19 increased the likelihood of organizational AI adoption (50.2%) and facilitation through investment (51.1%), training of healthcare workers (52.4%), and educating the public (54.2%). This suggests that ophthalmologists are unsure about the impact of the pandemic at the macrosystem level on influencing the willingness to implement clinical AI among service providers and organizations within the health system.

### Machine Learning Analysis for Clustering of Survey Responses

Feature permutation importance estimates were obtained to estimate the contribution of each feature, within the random forest model trained to predict each question outcome. For example, for the question “Will your organization be willing to adopt AI in clinical practice in 5 years,” the features corresponding to one's understanding of DL/ML, and ICO2017 income, were significantly more important than the others. Feature permutation importance involves randomly shuffling the values for a particular feature, and observing the decrease in model performance due to this shuffling. If the performance decreases appreciably, the feature is regarded as relatively predictive.

The AUCs of between 0.52 and 0.83 obtained in predicting binary outcomes with corresponding permutation importance suggest that eye care professionals' acceptance of AI and perceived likelihood of implementation can be anticipated from their demographics and attitudes toward AI to an extent (Appendix 2). The outcome variable predicted with the highest AUC of 0.83 was on whether AI could be an acceptable assistive tool for ophthalmologists, for which World Bank geographical region was the relatively most important predictor by permutation importance value. On the other hand, the AUC for the model in predicting acceptance for the application of AI as a diagnostic tool for ophthalmologists had relatively low values of 0.59 or below, which suggests the input variables in this study were not able to accurately predict ophthalmologists' acceptance of this application.

Responses to the APPRAISE survey indicated that participants were optimistic about the deployments of AI applications that reduce non-essential contact between patients and providers, thereby minimizing the risk of infectious disease transmission. This highlights the public health importance for further research and capacity building in this critical field to rapidly scale-up eye care services to meet the growing needs for eye screening in aging populations. Ophthalmologists will need to work closely with computer scientists to ensure that AI solutions for healthcare are appropriately designed to assimilate into clinical workflows and incorporate relevant considerations such as professional acceptance for specific AI applications based on the intended users for the given solution. Interestingly, participants from resource abundant settings reported potential barriers to adoption including lower odds of organizational willingness to adopt AI as well as lower acceptance of specific AI applications, including diagnostic tools for ophthalmologists that would need to be considered in developing specific applications. Although professional acceptance for AI solutions was relatively greater in resource-constrained settings, potential challenges with infrastructural availability such as intermittent electricity or internet access will need to be considered in the design of these solutions (7, 25).

### Limitations and Strengths

Limitations of this study include that a majority of participants originated from Asia Pacific, with less representation of participants from the West. Although the timing of survey dissemination facilitated evaluation of professional perspectives on AI adoption during an ongoing public health emergency, regions with higher official burden of COVID-19 at the time of survey dissemination had less representation in our results, as ophthalmologists may have been occupied with related public health initiatives at the time. Moreover, statistical assessment of the survey was not conducted and responses for all qualitative questions were not made compulsory, whereby non-response rates are indicated in the study tables. Therefore, description and analysis was conducted based on the valid responses with non-responses programmed as “missing.”

In addition, limitations of the snowball sampling method used for the purpose of hypothesis-generation in this study include the exclusion of other stakeholders such as primary care providers (PCPs). Furthermore, specific response rate calculation for different regions and channels for recruitment were not possible given privacy restrictions of the professional associations and inability to deconflict participants with membership in multiple associations. Although the survey was programmed to restrict one response per participant to avoid duplicate responses, there is potential selection bias for stakeholders that are more actively engaged in professional associations. These limitations may limit the generalisability of findings from this study. Future studies can address these limitations through survey validation for reliability and reproducibility, multiple testing correction for future hypothesis-testing research, probability sampling methods with inclusion of PCPs, stratified response rate tabulation based on individual channels of recruitment, and increased representation of participants from the West.

Finally, another limitation consistent with earlier survey investigations is the use of a logistic regression analytic approach to investigate associations between independent variables such as demographics and dependent variables such as acceptance, which assumes a linear relationship between them. However, unlike earlier investigations, one strength of this study is the use of a decision-tree based machine learning (ML) analytic approach called random forests to analyse responses in tandem with traditional logistic regression. The main distinction between the two analytic approaches rests in the transparency and underlying assumptions, whereby the flexibility of ML has allowed it to outperform the predictive accuracy of logistic regression in large empirical evaluations (53).

For logistic regression, a linear regression model on the input variables is transformed using the logistic function. It is therefore readily interpretable in terms of these input variables, assuming linearity of the input variables and log odds. However, logistic regression therefore remains a linear classifier, and non-linear relationships are not well-accounted for by the model. In contrast, the random forests ML classifier is able to model non-linear relationships in the data, with the trade-off of being less interpretable. This allows evaluation of a broader variety of potential relationships between the variables.

Additional strengths of this study include the consolidation of perspectives from a large and diverse spectrum of Ophthalmologists on the timely topic of clinical AI applications. The survey was also disseminated with close time-proximity to the COVID-19 outbreak, allowing assessment of the impact of a public health emergency on provider perspectives regarding clinical AI adoption. Finally, this study provides an in-depth investigation of professional acceptance of clinical AI solutions for automated classification of medical imaging in ophthalmology, incorporating a systematic approach to address factors affecting adoption at all levels from the micro-, meso-, and macrosystem. Furthermore, the intricacies of the latest regulatory guidance were applied in the evaluation of AI applications based on the intended user, significance of the information to the healthcare decision, and clinical context.

## Conclusion

Artificial Intelligence (AI) has been established as a tool for health systems to improve the right-siting of patients. This study outlines several key considerations that inform future research, communication and facilitation interventions to drive effective adoption and operationalization of these tools in clinical practice. Actionable insights to facilitate AI adoption are also highlighted, including engagement of relevant stakeholders and operationalization based on the enablers of AI adoption identified in this study, as well as addressing perceived barriers through development of the technology and guidelines in collaboration with ophthalmologists.

## Data Availability Statement

The datasets presented in this article are not readily available because aggregated de-identified data for participant responses in this study are provided in the segment on Supplementary Tables and Appendix. Informed consent was not obtained for sharing of individual responses (study data) outside of the preparation of this article. Requests to access the datasets should be directed to DG, mdcdvg@nus.edu.sg.

## Ethics Statement

The studies involving human participants were reviewed and approved by this research adhered to the tenets of the declaration of Helsinki, and Singhealth Institutional Review Board (IRB) approval was obtained with waiver of the need for informed consent (CIRB Ref 2020/2219). Written informed consent for participation was not required for this study in accordance with the national legislation and the institutional requirements.

## Author Contributions

DG, FZ, GL, CCYC, SZ, and WN contributed to the data analysis and drafting the initial manuscript. All listed authors contributed to study conceptualization, design, recruitment, data interpretation, manuscript preparation, and meet the criteria for authorship as agreed upon by the International Committee of Medical Journal Editors and are in agreement with the content of the manuscript. All authors contributed to the article and approved the submitted version.

## Conflict of Interest

DG reports appointment as Physician Leader (Telemedicine) for Raffles Medical Group (SGX:$BSL.SI) and investments in digital health start-ups AskDr, Doctorbell (acquired by MaNaDr), Shyfts, and VISRE. JC reports appointment as a consultant to Boston AI labs. AYL reports grants from Santen, personal fees from Genentech, US FDA, Johnson and Johnson, grants from Carl Zeiss Meditec, personal fees from Topcon, Gyroscope, non-financial support from Microsoft, grants from Regeneron, outside the submitted work; This article does not reflect the views of the US FDA. PK reports having acted as a consultant for DeepMind, Roche, Novartis, Apellis, and BitFount and is an equity owner in Big Picture Medical. He has received speaker fees from Heidelberg Engineering, Topcon, Allergan, and Bayer. AF reports honoraria, advisory board and grant funding from Alcon, Bayer, Novartis, Allergan, Roche, and Syneos Health. AL reports grants from Roche and Novartis, and appointment as a consultant to NotalVision, Allergan, Bayer, WebMD, and Beyeonics. AG reports appointment to provide lectures for Pfizer, Thea, and Polpharma. TS reports appointment as a consultant & advisory board for Bayer Yakuhin, Boehringer-Ingelheim, Novartis, Chugai, Senju, and Santen. DM reports funding support from the Singapore National Medical Research Council (NMRC-CIRG18Nov-0013), and the Duke-NUS Medical School, Singapore (ACP 05/FY2019/P2/06-A60). DM also reports appointment as consultant and Advisory Board Member of Optomed, Finland. TW reports appointment as the deputy group chief executive officer (research and education) of Singapore Health Services, a consultant & advisory board for Allergan, Bayer, Boehringer-Ingelheim, Genentech, Merck, Novartis, Oxurion (formerly ThromboGenics), Roche, and co-founder of Plano. DT reports funding from the following grants for research about AI in healthcare: National Medical Research Council, Singapore (NMRC/HSRG/0087/2018; MOH-000655-00), National Health Innovation Center, Singapore (NHIC-COV19-2005017), SingHealth Fund Limited Foundation (SHF/HSR113/2017), Duke-NUS Medical School, Singapore (Duke-NUS/RSF/2021/0018; 05/FY2020/EX/15-A58), and Agency for Science, Technology and Research (ASTAR), Singapore (A20H4g2141 and A20H4g2141). DT, GL, and TW also report being the co-inventors of a deep learning system for retinal diseases and co-founders of related start-up Eyris; potential conflicts of interests are managed according to institutional policies of the Singapore Health System (SingHealth) and the National University of Singapore (NUS). The remaining authors declare that the research was conducted in the absence of any commercial or financial relationships that could be construed as a potential conflict of interest.

## Publisher's Note

All claims expressed in this article are solely those of the authors and do not necessarily represent those of their affiliated organizations, or those of the publisher, the editors and the reviewers. Any product that may be evaluated in this article, or claim that may be made by its manufacturer, is not guaranteed or endorsed by the publisher.
