# Supplementary figures and images for: Acceptance and Perception of Artificial Intelligence Usability in Eye Care (APPRAISE) for Ophthalmologists: A Multinational Perspective
Source: Front Med (Lausanne). 2022 Oct 13;9:875242. doi: 10.3389/fmed.2022.875242 (PMC9612721; doi:10.3389/fmed.2022.875242)

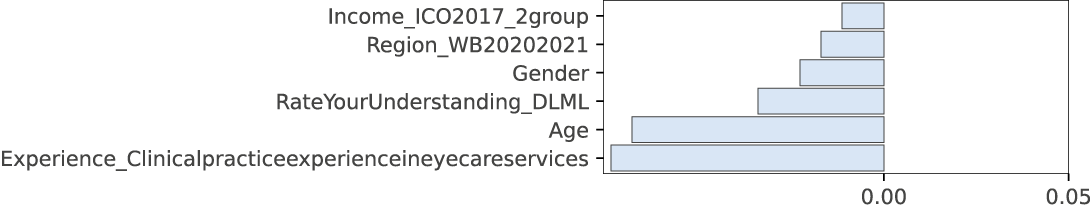

Supplement: Supplementary file 1 [file Data_Sheet_1.ZIP › APPRAISE_6variables_featimp_20220302/rfpimp_Acceptable_AssistiveToolForOphthalmologists.png]

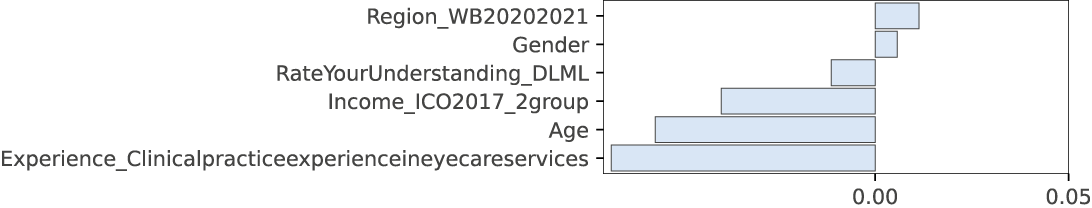

Supplement: Supplementary file 1 [file Data_Sheet_1.ZIP › APPRAISE_6variables_featimp_20220302/rfpimp_Acceptable_AssistiveToolForPrimaryEyeCareProviders.png]

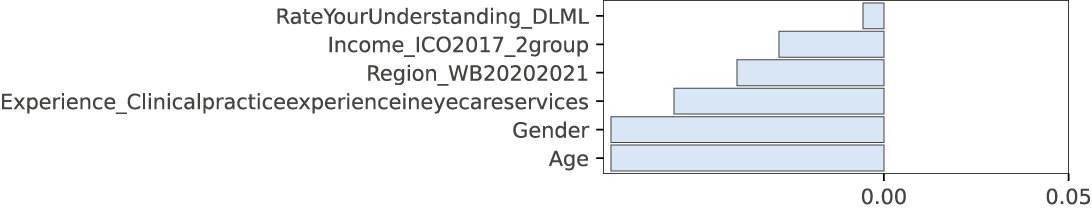

Supplement: Supplementary file 1 [file Data_Sheet_1.ZIP › APPRAISE_6variables_featimp_20220302/rfpimp_Acceptable_CDSToolForOphthalmologists.png]

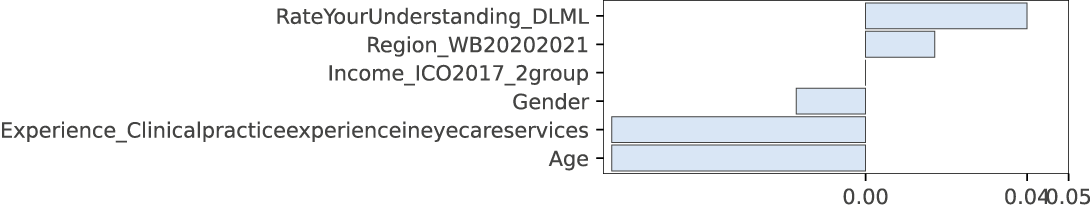

Supplement: Supplementary file 1 [file Data_Sheet_1.ZIP › APPRAISE_6variables_featimp_20220302/rfpimp_Acceptable_CDSToolForPrimaryEyeCareProviders.png]

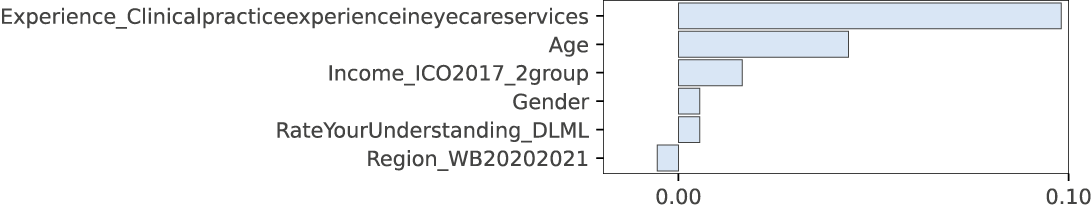

Supplement: Supplementary file 1 [file Data_Sheet_1.ZIP › APPRAISE_6variables_featimp_20220302/rfpimp_Acceptable_DiagnosticToolForOphthalmologists.png]

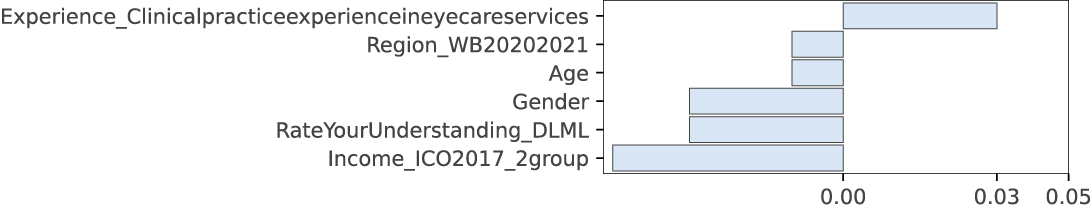

Supplement: Supplementary file 1 [file Data_Sheet_1.ZIP › APPRAISE_6variables_featimp_20220302/rfpimp_Acceptable_DiagnosticToolForPrimaryEyeCareProviders.png]

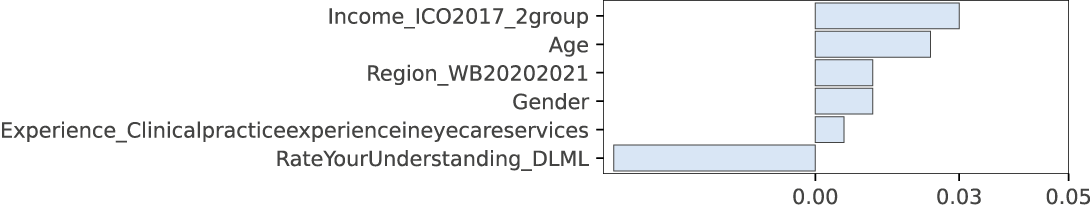

Supplement: Supplementary file 1 [file Data_Sheet_1.ZIP › APPRAISE_6variables_featimp_20220302/rfpimp_OrganisationAIAMDdiagnosis.png]

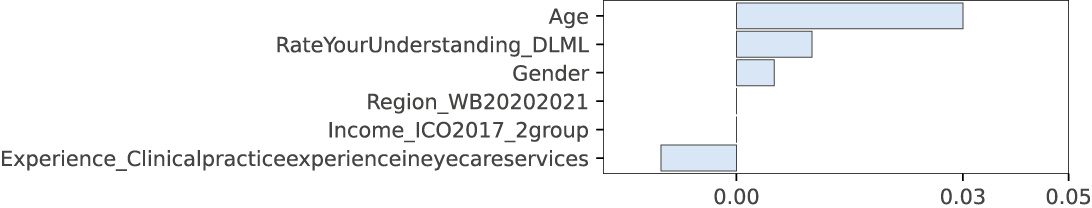

Supplement: Supplementary file 1 [file Data_Sheet_1.ZIP › APPRAISE_6variables_featimp_20220302/rfpimp_OrganisationAIAMDscreen.png]

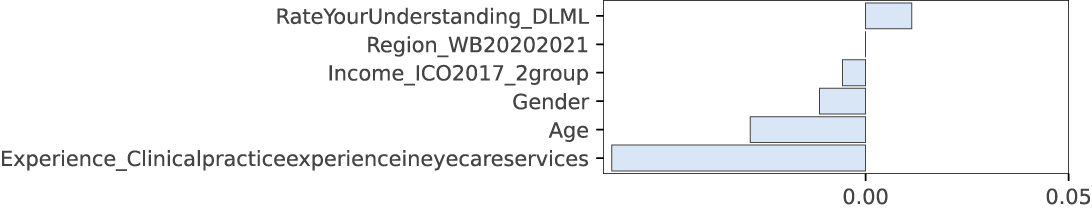

Supplement: Supplementary file 1 [file Data_Sheet_1.ZIP › APPRAISE_6variables_featimp_20220302/rfpimp_OrganisationAICataractdiagnosis.png]

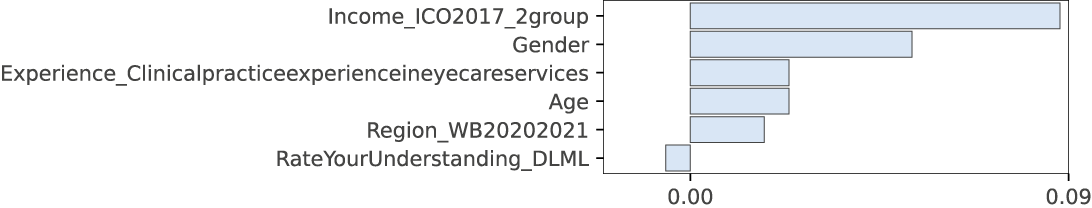

Supplement: Supplementary file 1 [file Data_Sheet_1.ZIP › APPRAISE_6variables_featimp_20220302/rfpimp_OrganisationAICataractscreen.png]

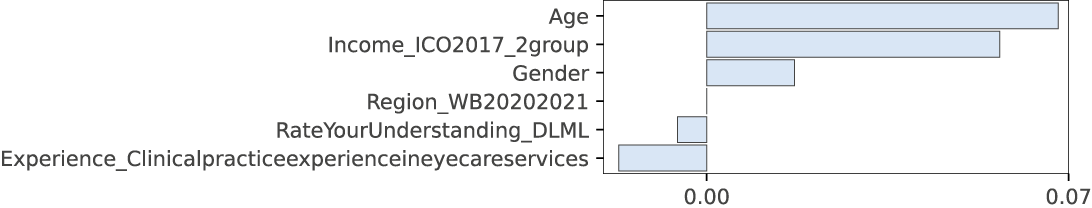

Supplement: Supplementary file 1 [file Data_Sheet_1.ZIP › APPRAISE_6variables_featimp_20220302/rfpimp_OrganisationAIDRdiagnosis.png]

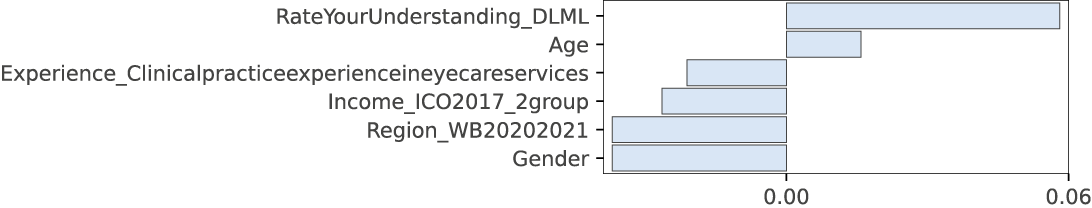

Supplement: Supplementary file 1 [file Data_Sheet_1.ZIP › APPRAISE_6variables_featimp_20220302/rfpimp_OrganisationAIDRscreen.png]

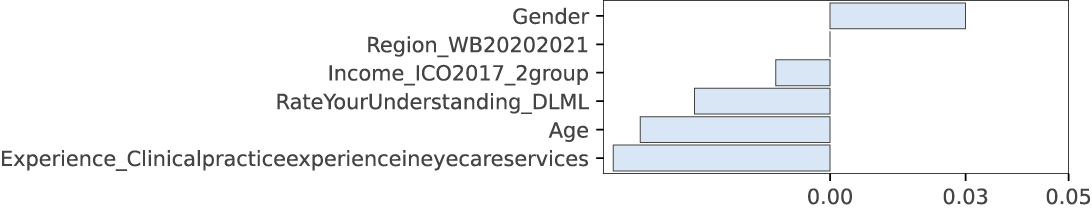

Supplement: Supplementary file 1 [file Data_Sheet_1.ZIP › APPRAISE_6variables_featimp_20220302/rfpimp_OrganisationAIGlaucomadiagnosis.png]

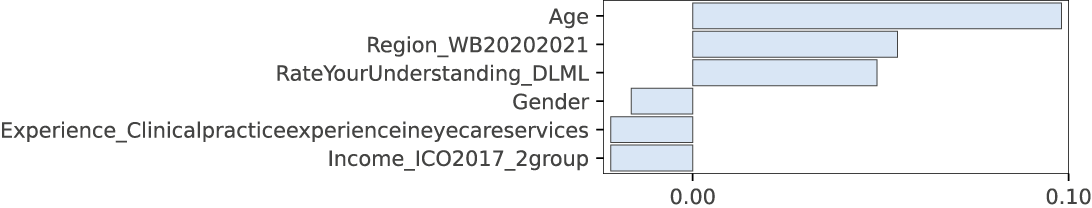

Supplement: Supplementary file 1 [file Data_Sheet_1.ZIP › APPRAISE_6variables_featimp_20220302/rfpimp_OrganisationAIGlaucomascreen.png]

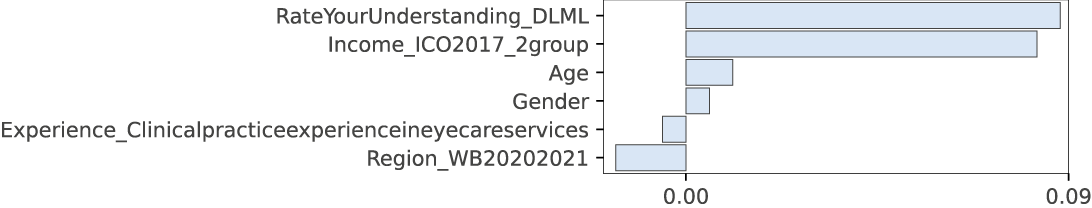

Supplement: Supplementary file 1 [file Data_Sheet_1.ZIP › APPRAISE_6variables_featimp_20220302/rfpimp_WillyourorganisationbewillingtoadoptAIinclinicalpracticein5yrs-01.png]
