# Supplementary material for: Acceptance and Perception of Artificial Intelligence Usability in Eye Care (APPRAISE) for Ophthalmologists: A Multinational Perspective
Source: Front Med (Lausanne). 2022 Oct 13;9:875242. doi: 10.3389/fmed.2022.875242 (PMC9612721; doi:10.3389/fmed.2022.875242)
Supplement: Supplementary file 2 [file Table_1.DOCX]

**Acceptance and Perspective of AI Usability in Eyecare During and Beyond COVID-19: Survey questions**

Q1) Current area of practice

Q2) Clinical practice experience in eye care services (Eye screening, Optometry, Ophthalmology, etc)

Q3) In which country do you Practice?

Q4) Gender

Q5) Age

Q6) How would you rate your understanding about deep learning, machine learning, and AI?

Q7) Will your organisation be willing to adopt AI in clinical practice in the next 5 years?

Q8) Which area(s) is your organisation likely to implement AI for in the next 5 years? (Yes, No, Unsure for each)

Q9) How long do you think it will be before AI is regularly used in clinical practice for Ophthalmology in your organisation?

Q10) How will AI affect Ophthalmology clinical workload in your organisation beyond the next decade?

Q11) How should your organisation adjust ophthalmology trainee numbers in the next 5 years in preparation for the deployment of AI in ophthalmology?

Q12) How should your organisation adjust the number eye care allied health trainee (optometrists, orthoptists etc) numbers in the next 5 years in preparation for the deployment of AI in ophthalmology?

Q13) From your point of view, is your organisation likely to train healthcare workers in the use and understanding of AI in the next 5 years?

Q14) From your point of view, Is your organisation likely to invest resources, such as finances and personnel, to implement AI in Ophthalmology in the next 5 years?

Q15) From your point of view, Is your organisation likely to be active in educating the public regarding the use of AI in Ophthalmology in the next 5 years?

Q16) This question relates to the acceptability of AI for primary eye care providers (GP, Optometrists, etc) or Ophthalmologists in future, once clinically acceptable classification accuracy is established. Three potential use cases include:

- as an **assistive too**l: AI is used to highlight areas of interest in ophthalmic images for the practitioner. The practitioner then considers this information together with their clinical findings.
- as a **clinical decision support** (CDS) too: AI is used to highlight areas of interest on ophthalmic images, provides a provisional diagnosis. The practitioner then considers this information together with their clinical findings.
- as a **diagnostic tool** (fully automated): AI provides the clinical diagnosis, including the stage of disease, autonomously with or without management suggestions. The practitioner then considers this information with or without undertaking a clinical examination.

Do you agree that these applications of AI are acceptable?

(i) Assistive tool for primary eye care providers

(ii) CDS tool for primary eye care providers

(iii) Diagnostic tool for primary eye care providers

(iv) Assistive tool for Ophthalmologists

(v) CDS tool for Ophthalmologists

(vi) Diagnostic tool for Ophthalmologists

Q17) This question relates to the acceptable level of error (e.g. false negative/positive) for applications of AI described in the previous question.

What level of error is acceptable?

(i) Assistive tool for primary eye care providers

(ii) CDS tool for primary eye care providers

(iii) Diagnostic tool for primary eye care providers

(iv) Assistive tool for Ophthalmologists

(v) CDS tool for Ophthalmologists

(vi) Diagnostic tool for Ophthalmologists

Q18) Which of these are potential advantages for the use of AI systems in ophthalmology? (options for each are: Yes, No, Unsure)

(i) Improved patient access to disease screening

(ii) More targeted referrals to specialist medical care

(iii) Reduced time spent by specialists on monotonous tasks

(iv) Improved diagnostic confidence

(v) Improved prediction of disease outcomes

(vi) More personalised and evidence-based disease management

(vii) More cost-effective health care

(vii) Greater uniformity in diagnosis and management decisions

Q19) Do you consider any of these as concerns/ drawbacks for the application of AI in ophthalmology? (options for each are: Yes, No, Unsure)

(i) Impact on workforce needs

(ii) Concerns over benchmarking clinicians against machines

(iii) Challenge to the fiduciary relationship between patient and doctor

(iv) Data security & privacy concerns

(v) Concerns over medical liability due to machine error

(vi) Lack of confidence or trust in “black-box” diagnosis

(vii) Decreasing reliance on medical specialists for diagnosis and treatment advice

(vii) Concerns over the divestment of health care to large technology and data companies

Q20) The eye care roles and responsibilities of which professional group(s) will be replaced by AI within the next 5 years?

(Options: Yes Totally replaced, Yes Partially replaced, No not replaced, or Unsure)

(i) Primary eye care services (Optometrists, Opticians, etc)

(ii) Ophthalmologist

(iii) Primary care provider with eye care services (e.g. General Practitioner)

Q21) Do you agree with the following statements?

(i) ACCESSIBILITY of eye care will improve with the introduction of AI

(ii) AFFORDABILITY of eye care will improve with the introduction of AI

(iii) QUALITY of eye care will improve with the introduction of AI

Q22) 2020 saw the emergence of the coronavirus disease 2019 (COVID-19) pandemic, whereby many front-line eye care professionals were exposed to the infection for many reasons, including close proximity during ocular examination and high clinical load of outpatient services.

Having had this experience, do you agree with the following statements?

(i) AI can reduce non-essential contact between providers and patients.

(ii) My organisation is more likely to adopt AI in the next 5 years.

(iii) My organisation is more likely to train healthcare workers in the use of AI in the next 5 years.

(iv) My organisation is more likely to invest resources to implement AI in Ophthalmology in the next 5 years.

(v) My organisation is more likely to be active in educating the public regarding the use of AI in Ophthalmology in the next 5 years.
